# Reduction of fatigue and anger-hostility by the oral administration of 5-aminolevulinic acid phosphate: a randomized, double-blind, placebo-controlled, parallel study

**DOI:** 10.1038/s41598-020-72763-4

**Published:** 2020-09-29

**Authors:** Fumiko Higashikawa, Keishi Kanno, Akiko Ogata, Masanori Sugiyama

**Affiliations:** 1 Department of Probiotic Science for Preventive Medicine, Graduate School of Biomedical and Health Sciences, Hiroshima University, Kasumi 1-2-3, Minami-ku, Hiroshima, 734-8551 Japan; 2 Department of General Internal Medicine, Hiroshima University Hospital, 1-2-3 Kasumi, Minami-ku, Hiroshima, Hiroshima 734-8551 Japan; 3 Department of Psychology, Graduate School of Education, Hiroshima University, 1-1-1 Kagamiyama, Higashi-Hiroshima, Hiroshima 739-8511 Japan

**Keywords:** Human behaviour, Nutrition

## Abstract

Although large populations feel fatigue, the standardized medicinal therapy is currently absent. In this study, we determined whether 5-aminolevulinic acid (5-ALA) supplementation alleviates the feeling of fatigue in healthy subjects who feel chronic physical tiredness. Males and females between ages of 20 and 64 who felt physical fatigue on a daily basis, with a visual analogue scale (VAS) for fatigue ≥ 40 mm, a T-score of Fatigue-Inertia in the Profile of Mood States—Second Edition—Adult (POMS2-A) ≥ 50, and a T-score of Vigor-Activity in POMS2-A ≤ 60 were recruited. Seventy eligible participants were randomly assigned to either a 5-ALA or a placebo group. During the 8 weeks of consumption, the subjects completed VAS questionnaires for fatigue and POMS2-A at 4-week intervals. The VAS values for overall feeling of fatigue and feeling of work-related fatigue, and the Anger-Hostility subscale of POMS2-A were decreased by 5-ALA with significant time × group interaction effects (*p* = 0.040, 0.020, and 0.045, respectively). Besides, the 5-ALA group showed significant differences in Fatigue-Inertia, Depression-Dejection and Total Mood Disturbance scores, when compared between pre- and post-intervention, while the placebo group did not. In conclusion, the oral administration of 5-ALA improves fatigue and negative mood in subjects who constantly feel physical fatigue.

This clinical trial was registered with University hospital Medical Information Network Clinical Trials Registry (UMIN-CTR) as UMIN000031528 on 2/3/2018.

## Introduction

Fatigue is a commonly occurring health condition in any person, and the excessive or chronic fatigue can lead to various diseases. The prevalence of fatigue is considerably high and is one out of three to five people in general population^1,2^. Since there is no standardized medicinal therapy to break free from fatigue, it is better to have adequate nutrients, good-quality sleep, enough rest, and functional foods if desired to avoid fatigue. Nevertheless, chronic fatigue that cannot to be explained by any underlying medical condition may be difficult to manage. In the Japanese market, several dietary supplements, such as γ-aminobutyric acid^3^, coenzyme Q10^4^, and citric acid^5^, are sold claiming to reduce fatigue.

5-Aminolevulinic acid (5-ALA), which is a precursor of heme, is the first compound produced in the heme synthetic pathway. 5-ALA is contained in various foods, including vegetables, fruits, and fermented liquors. Heme is an essential component of hemoglobin, myoglobin, cytochromes, and drug metabolism enzymes—cytochrome P450s. It has been reported that 5-ALA supplementation activates the final enzyme in the mitochondrial electron transfer chain, cytochrome *c* oxidase^6^. To understand the biological importance of heme protein, various energy-related effects are expected by the administration of 5-ALA. To date, 5-ALA supplementation has been shown to have possible applications in type 2 diabetes mellitus^7,8^ and/or Alzheimer’s disease^9^. Since it has been previously reported that 5-ALA improved mood and coping ability in middle-aged and older adults with prediabetes in randomized study^10^, 5-ALA may be a candidate for managing fatigue through the activation of mitochondrial energy metabolism.

In this study, we evaluated the effects of 5-ALA supplementation on chronic fatigue and mood states in a randomized, double-blind, placebo-controlled study.

## Results

**Figure 1:**
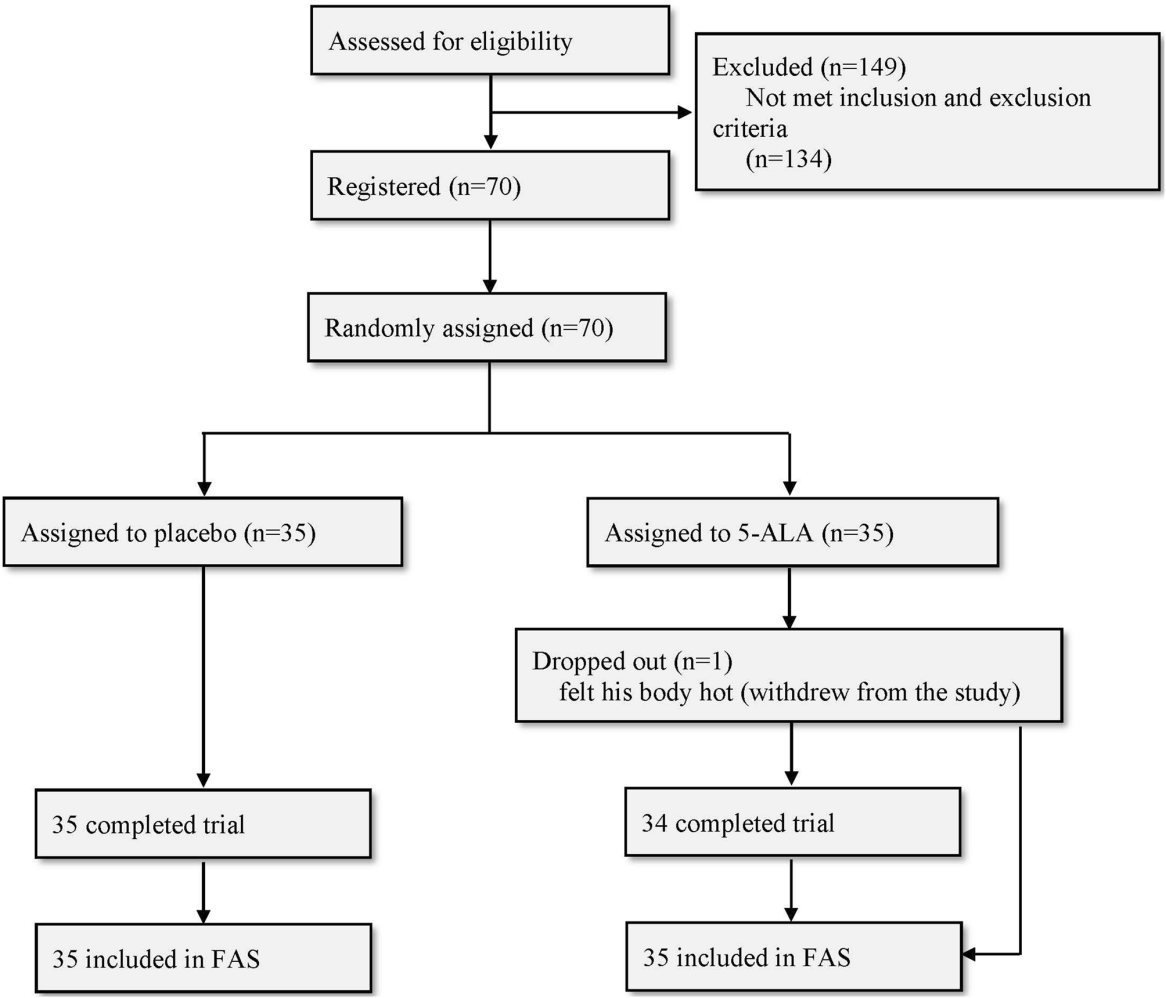
Summary of the subject flow.

Of 219 applicants, 149 were excluded because they did not meet the criteria or withdrew from participation. Seventy individuals were registered and randomized. The participants were all Japanese. One subject in the 5-ALA group dropped out after the third visit (week 4) complaining that his body felt hot during the intake (Fig. 1). The missing data were filled in by multiple imputation, and then all participants were subjected to statistical analyses.

The background of study participants is summarized in Table 1. There were no statistical differences between the two groups in all of the variables listed in Table 1 at the baseline. No differences in compliance (97.8 ± 2.8% in the placebo group and 94.0 ± 8.8% in the 5-ALA group) or change in calorie intake were detected between the groups (data not shown).

**Table 1:** Baseline characteristics of the study participants.

	Placebo (n = 35)	5-ALA (n = 35)
Age (years)	50.4 ± 9.9^a^	51.2 ± 10.1
**Sex [n (%)]**		
Male	9 (25.7)	10 (28.6)
Female	26 (74.3)	25 (71.4)
Height (m)	1.61 ± 0.08	1.59 ± 0.07
Body weight (kg)	56.4 ± 13.0	56.4 ± 10.2
BMI (kg/m^2^)	21.7 ± 3.8	22.3 ± 3.0
Body fat percentage (%)	25.9 ± 7.7	26.3 ± 7.0
Systolic blood pressure (mmHg)	124 ± 19	121 ± 18
Diastolic blood pressure (mmHg)	76.4 ± 13.6	72.8 ± 13.5
Heart rate (beats per min)	68.9 ± 11.3	66.2 ± 10.2
White blood cell count (× 10^3^/mL)	5.23 ± 1.54	5.13 ± 1.41
Red blood cell count (× 10^6^/mL)	4.67 ± 0.47	4.64 ± 0.40
Hemoglobin (g/dL)	13.9 ± 1.7	13.8 ± 1.5
Hematocrit (%)	42.8 ± 4.1	42.8 ± 4.1
Platelet count (× 10^4^/mL)	27.2 ± 6.0	26.0 ± 7.0
AST (IU/L)	21.8 ± 5.3	22.2 ± 5.2
ALT (IU/L)	18.3 ± 8.3	21.0 ± 10.6
γ-GTP (IU/L)	29.1 ± 27.8	29.6 ± 28.0
LDH (IU/L)	187 ± 29	183 ± 29
Choline esterase (IU/L)	340 ± 63	332 ± 61
Alkaline phosphatase (IU/L)	206 ± 54	208 ± 59
Amylase (IU/L)	86.1 ± 32.5	84.5 ± 27.5
Total protein (g/dL)	7.56 ± 0.46	7.46 ± 0.39
Total bilirubin (mg/dL)	0.69 ± 0.30	0.67 ± 0.35
Albumin (g/dL)	4.59 ± 0.32	4.51 ± 0.25
Uric acid (mg/dL)	4.79 ± 1.24	4.84 ± 1.17
Blood urea nitrogen (mg/dL)	12.7 ± 3.6	13.5 ± 3.6
Creatinine (mg/dL)	0.70 ± 0.16	0.70 ± 0.13
eGFR (mL/min/1.73 m^2^)	98.4 ± 21.0	97.8 ± 21.6
Total cholesterol (mg/dL)	221 ± 37	224 ± 43
LDL cholesterol (mg/dL)	128 ± 30	138 ± 40
HDL cholesterol (mg/dL)	76.8 ± 22.1	72.9 ± 16.8
LDL/HDL ratio	1.82 ± 0.69	2.01 ± 0.84
Triglycerides (mg/dL)	109 ± 129	87 ± 62
Fasting blood glucose (mg/dL)	97.6 ± 7.3	98.0 ± 6.7

^a^Mean ± SD for all such values.

As the primary endpoint, visual analogue scale (VAS) value for overall feeling of fatigue (VAS-1) decreased 14.4 mm by 5-ALA supplementation with a significant time × group interaction effect (*p* = 0.040, Table 2). VAS value for feeling of work-related fatigue (VAS-2) was also significantly diminished with 13.3 mm in the 5-ALA group (*p* = 0.020 in time × group interaction, Table 2). When compared between pre- and post-intervention (baseline and 8 weeks), all VAS values in the 5-ALA group were significantly reduced after intervention but not in the placebo group (Table 2).

**Table 2:** Changes in the visual analogue scale (VAS) for fatigue after 5-ALA administration.

	Placebo (n = 35)	5-ALA (n = 35)	F-statistic (df1, df2)^a^ *p*^a^		
			Time	Group	Time × group
**VAS value for overall feeling of fatigue (VAS-1)**			1.491 (1.791,67)	0.162 (1,67)	3.456 (1.791,67)
Baseline	66.0 ± 18.6^b^	73.0 ± 15.9	0.283	0.688	0.040
Week 4	64.1 ± 17.9	62.9 ± 17.7			
Week 8	62.6 ± 22.6	58.6 ± 21.6^†††^			
Change in 8 weeks	− 3.4 ± 27.5	− 14.4 ± 19.2			
**VAS value for feeling of work-related fatigue (VAS-2)**			2.012 (2,67)	2.048 (1,67)	4.035 (2,67)
Baseline	64.3 ± 19.7	75.2 ± 12.3	0.143	0.157	0.020
Week 4	64.0 ± 19.8	64.3 ± 17.2			
Week 8	61.8 ± 21.3	61.9 ± 21.2^†††^			
Change in 8 weeks	− 2.5 ± 26.1	− 13.3 ± 16.7*			
**VAS value for efficiency at work (VAS-3)**			1.116 (2,67)	0.457 (1,67)	0.118 (2,67)
Baseline	62.8 ± 19.5	69.6 ± 18.4	0.341	0.502	0.118
Week 4	60.7 ± 21.0	58.3 ± 20.6			
Week 8	58.3 ± 20.8	60.9 ± 23.6^†^			
Change in 8 weeks	− 4.5 ± 25.2	− 8.7 ± 20.2			
**VAS value for feeling of fatigue when wake-up in the morning (VAS-4)**			1.274 (2,67)	0.007 (1,67)	0.963 (2,67)
Baseline	66.3 ± 18.5	69.0 ± 19.4	0.297	0.934	0.384
Week 4	61.4 ± 21.3	60.0 ± 21.4			
Week 8	59.0 ± 22.0	57.1 ± 27.0^†††^			
Change in 8 weeks	− 7.4 ± 23.5	− 11.9 ± 18.4			

*VAS* visual analogue scale.

^†^,^†††^Statistical difference versus baseline by paired t-test at *p* < 0.05 or *p* < 0.005, respectively.

*Statistical difference versus placebo by student t-test at *p* < 0.05.

^a^Two-way repeated measures ANOVA.

^b^Mean ± SD for all such values.

The average T-scores of all negative mood indices in Profile of Mood States-Second Edition for Adult (POMS2-A)—including FI (Fatigue-Inertia), AH (Anger-Hostility), CB (Confusion-Bewilderment), DD (Depress-Dejection), TA (Tension-Anxiety), and TMD (Total Mood Disturbance)—decreased; however, only AH showed significant difference in time × group interaction (Table 3). The T-scores of FI, AH, DD, and TMD were significantly decreased only in the 5-ALA group, when comparing before and after the intake period. The positive mood indices, such as VA (Vigor-Activity) and F (Friendliness), were increased in both intervention groups without significant differences in the 8-week changes between the groups.

**Table 3:** Changes in each Profile of Mood States 2nd Edition—adult (POMS2-A) subscale.

	Placebo (n = 35)	5-ALA (n = 35)	F-statistic (df1, df2)^a^ *p*^a^		
			Time	Group	Time × group
**Fatigue-Inertia (FI)**			1.514 (2,67)	2.758 (1,67)	1.590 (2,67)
Baseline	57.5 ± 9.2^b^	62.1 ± 10.2	0.356	0.101	0.208
Week 4	57.6 ± 9.3	59.6 ± 11.0			
Week 8	55.7 ± 8.0	56.9 ± 12.9^†^			
Change in 8 weeks	− 1.8 ± 9.5	− 5.2 ± 12.3			
**Vigor-Activity (VA)**			0.745 (2,67)	1.871 (1,67)	0.965 (2,67)
Baseline	45.3 ± 7.2	43.2 ± 5.6	0.548	0.176	0.384
Week 4	47.8 ± 8.2	45.2 ± 7.6			
Week 8	47.9 ± 7.4^†^	47.0 ± 7.2^†††^			
Change in 8 weeks	2.6 ± 6.8	3.9 ± 6.0			
**Anger-Hostility (AH)**			0.863 (2,67)	3.515 (1,67)	3.164 (2,67)
Baseline	51.5 ± 8.9	58.5 ± 13.3	0.431	0.065	0.045
Week 4	53.7 ± 9.5	56.8 ± 12.2			
Week 8	51.1 ± 6.2	53.0 ± 10.4^†††^			
Change in 8 weeks	− 0.4 ± 8.7	− 5.4 ± 10.3*			
**Confusion-Bewilderment (CB)**			0.696 (1.808,67)	0.194 (1,67)	0.069 (1.808,67)
Baseline	53.6 ± 8.7	56.4 ± 12.3	0.528	0.194	0.918
Week 4	53.1 ± 9.1	55.5 ± 12.1			
Week 8	52.5 ± 8.6	55.1 ± 12.8			
Change in 8 weeks	− 1.2 ± 7.5	− 1.2 ± 9.9			
**Depression-Dejection (DD)**			0.434 (2,67)	2.085 (1,67)	1.300 (2,67)
Baseline	54.9 ± 10.0	59.2 ± 15.0	0.667	0.153	0.276
Week 4	53.4 ± 9.4	58.9 ± 13.9			
Week 8	53.5 ± 8.2	56.2 ± 12.8^†^			
Change in 8 weeks	− 1.4 ± 8.9	− 3.0 ± 8.3			
**Tension-Anxiety (TA)**			2.581 (1.516,67)	2.772 (1,67)	0.756 (1.516,67)
Baseline	56.0 ± 8.3	60.0 ± 11.7	0.400	0.102	0.446
Week 4	55.4 ± 7.9	58.7 ± 10.5			
Week 8	55.5 ± 8.4	57.8 ± 14.2			
Change in 8 weeks	− 0.5 ± 10.4	− 2.2 ± 13.0			
**Friendliness (F)**			0.501 (2,67)	0.024 (1,67)	0.389 (2,67)
Baseline	46.8 ± 8.4	46.0 ± 7.9	0.677	0.878	0.679
Week 4	49.3 ± 8.5	50.0 ± 7.3			
Week 8	52.0 ± 10.1^†††^	52.6 ± 9.7^†††^			
Change in 8 weeks	5.2 ± 7.3	6.6 ± 6.7			
**Total Mood Disturbance (TMD)**			1.227 (1.713,67)	3.180 (1,67)	1.548 (1.713,67)
Baseline	55.8 ± 8.9	61.0 ± 12.9	0.381	0.079	0.219
Week 4	55.1 ± 9.0	59.4 ± 12.2			
Week 8	54.1 ± 7.4	56.8 ± 13.0^†^			
Change in 8 weeks	− 1.7 ± 8.2	− 4.2 ± 10.1			

^†^^,†††^Statistical difference versus baseline by paired *t* test at *p* < 0.05 or *p* < 0.005, respectively.

*Statistical difference versus placebo by Student *t* test at *p* < 0.05.

^a^Two-way repeated measures ANOVA.

^b^Mean ± SD for all such values.

Except for the participant who withdrew from the study, no obvious adverse events that were possibly related to 5-ALA consumption were detected, based on physiological examination, hematological assessment, biochemical examination of the blood, urine testing, and subjective symptoms.

## Discussion

In this study, we evaluated the effects of 5-ALA on fatigue and mood states. To convert the subjective level of feeling of fatigue into objective data, VAS and POMS2-A were employed. VAS was originally used as a pain scale; however, it is also useful for other subjective estimates. VAS is now utilized as a tool to assess fatigue in many areas of clinical research^4,5,11–13^.

Time × group interaction effects were observed in VAS-1, the overall feeling of fatigue, and in VAS-2, the feeling of work-related fatigue. 5-ALA administration reduced 14.4 mm in VAS-1 and 13.3 mm in VAS-2. Additionally, within-group differences were observed in VAS values for efficiency at work (VAS-3) and for feeling of fatigue when wake-up in the morning (VAS-4) as well (*P* < 0.05 and *P* < 0.001 vs. each baseline, respectively). Judging from these results holistically, 5-ALA improves overall fatigue, reduces work-related fatigue, increases work efficiency, and lessens fatigue when waking up.

POMS is a self-report assessment of mood and indicates transient, fluctuating feelings and enduring affect states. POMS is also used in various research fields to measure mood disturbance and/or fatigue involved in cancer, stroke, multiple sclerosis, or menopausal disorder, in addition to clinical usage in the department of psychiatry^14–17^.

According to the manual of POMS2 described in Japanese^18^, the higher the T-scores in AH, CB, DD, FI, and TA, the more negative the mood and/or mood disturbance indicated. In positive mood states—VA and F, higher T-scores suggest feeling better. T-scores are standardized, as the average is 50 with an SD of 10.

In this clinical trial, T-score of AH (Anger-Hostility) were significantly decreased in the 5-ALA group (*P* = 0.045 in time × group interaction). The characteristics of individuals with high AH T-scores include tendencies to easily become irritated, feel bad, develop a sense of aversion, or express outrage^18^. In all 11 POMS2-A questionnaires related to AH, in a total of 65, the mean degrees of reduction were larger in the 5-ALA group than in the placebo group (data not shown). This result also supports that 5-ALA administration has beneficial effects in reducing negative moods with anger and hostility.

On the other hand, FI did not show a significant decrease in the 5-ALA group based on a between-group comparison. Nonetheless, we presumed that 5-ALA positively affects FI for two reasons: a within-group difference was observed only in the 5-ALA group, and the scores of 6 questionnaires related to FI in POMS2-A were all reduced to a greater degree in the 5-ALA group than in the placebo group (data not shown). According to the within-group analyses, DD and TMD declined in the 5-ALA group (both *P* < 0.05 vs. each baseline), VA and F were increased in both groups, and CB and TA showed no changes in both groups. These trends in the POMS2-A results are consistent with the VAS results.

Two studies in different countries reported that approximately 22% and 38% of people experience fatigue in the general population^1,2^. Despite fatigue is the most common complaints in primary care practice, there is currently no effective therapy for fatigue of uncertain cause. Vitamins/mineral supplementation may help to reduce stress and improve mental health and cognitive performance^19^. It has also been suggested that BCAA, a beverage combining arginine and carbohydrate, improves exercise recovery^20^. Moreover, the administration of coenzyme Q10 or citric acid improves fatigue^4,5^. Thus, the administration of appropriate dietary supplements might be helpful for managing daily fatigue.

5-ALA is a natural amino acid that is widely distributed in both animals and plants. The substance is necessary for the biosynthesis of heme and chlorophyll. Heme is an essential component in mitochondrial respiratory chain reaction as heme proteins. In terms of this importance of heme proteins for energy production, its precursor—5-ALA—might be considered as a source of life. Therefore, it is not surprising that the intake of 5-ALA can reduce fatigue via the mitochondrial electron transport system, activating cytochrome *c* oxidase^6^ and enhancing ATP production. Indeed, mitochondrial disorders are defects in cellular energy, and exhibit various symptoms including fatigue, skeletal muscle weakness, exercise intolerance and so on^21^. Previous study has suggested that 6 weeks 5-ALA administration improved sleep quality in subjects with insomnia or difficulty sleeping, reducing the Pittsburgh Insomnia Rating Scale-20 Question (PIRS-20)^22^. In addition, 5-ALA improved exercise efficiency and home-based walking training achievement in older women^23^, and also improved depressive symptoms measured by the Montgomery–Åsberg Depression Rating Scale (MADRS) in middle-aged depressive women^24^. These evidences are consistent with our results that 5-ALA improves physical tiredness and negative mood.

It has been reported that the environmental conditions, such as temperature, humidity, and sunshine duration, influence people’s mental health^25,26^. The season when this study was conducted was early summer and the rainy season in Hiroshima. In the study, all participants in both intervention groups spent the experimental period under the quite similar environmental condition, since they all lived in and around Hiroshima and they were assessed within a narrow range—17 days difference at maximum—for each clinical visit. Therefore, the seasonal influence could be eliminated from the data interpretation.

In conclusion, in this clinical trial, it was shown that the oral administration of 5-ALA improved fatigue and negative feelings—anger-hostility. These results demonstrate that 5-ALA may be useful for keeping people well physically and mentally in such a stressful modern society.

## Methods

### Materials

An experimental tablet containing 10 mg of 5-ALA phosphate and 11.5 mg of sodium ferrous citrate (SFC) was provided by SBI ALApromo Co., Ltd., Tokyo, Japan. A placebo tablet, containing 11.5 mg of sodium ferrous citrate without 5-ALA phosphate, was also provided by the same company.

### Subjects

Participants for the clinical trial were recruited from the Hiroshima area of Japan via advertisement. The inclusion criteria of the study were as follows: (1) healthy volunteers between 20 and 64 years of age who (2) felt physical fatigue on a day-to-day basis (3) with > 40 mm on the visual analogue scale (VAS) for overall feeling of fatigue (4) with ≥ 50 T-score in fatigue-inertia and ≤ 60 T-score in Vigor-Activity of POMS2-A subscales. T-score was calculated using the T-score conversion table of each age group and gender. Participants with any of the following conditions were excluded: (1) taking medicines for chronic disease; (2) taking functional foods that might affect the trial; (3) having participated in any clinical trial within 90 days of the commencement of the trial; (4) being pregnant or nursing a child; or (5) being judged as ineligible by clinical investigators. The study protocol was approved by the Ethics Committee of Hiroshima University and performed in accordance with the guidelines of the Helsinki Declaration. Written informed consent was obtained from each participant before enrolling in the study.

### Study design

This clinical study was conducted as a randomized, double-blind, placebo-controlled, parallel trial at Hiroshima University Hospital, from February 2018 to July 2018. Seventy subjects enrolled by a study investigator were randomly assigned to either the 5-ALA group or the placebo group with a 1:1 allocation ratio using the block randomization method with block size of four. The allocation sequence was generated using a computer software program by a non-clinical staff member who was not the data analyzer. The same non-clinical staff assigned participants to interventions. The subjects and outcome assessors were blinded to the randomization assignment.

The subjects received 3 tablets/day containing either a total of 30 mg of 5-ALA + 34.5 mg SFC (3.6 mg as Fe) or only 34.5 mg SFC at any time during the day for 8 weeks.

Subjects were instructed to: (1) maintain their ordinary daily life without taking excess calories and/or alcohol during the study; (2) record whether they take the tablets, health conditions, medicines and supplements taken, and unusual life events; (3) record meal contents, including snacks and alcohol, for 7 days before the clinical visit; (4) to refrain from donating blood.

VAS questionnaires consisted of four questions about the overall feeling of fatigue (VAS-1), feeling of work-related fatigue (VAS-2), efficiency at work (VAS-3), and feeling of fatigue when waking up in the morning (VAS-4). For each question, the subjects intuitively marked their responses on the 100 mm line, considering their fatigue levels over the last week. For VAS-1, 0 mm was considered as experiencing no fatigue at all and 100 mm as experiencing the worst fatigue so that they were unable to do anything. Likewise, 0 mm was considered as experiencing no work-related fatigue at all, the state they could most efficiently work without any stress, or experiencing no fatigue at all when waking up in the morning, for VAS-2, VAS-3, or VAS-4, respectively. Moreover, 100 mm was considered as experiencing the worst fatigue so that they were unable to do anything due to work, the state in which they could not work at all due to fatigue, or experiencing the worst fatigue so that they unable to do nothing when waking up in the morning, for VAS-2, VAS-3, or VAS-4, respectively. Work-related fatigue included that experienced due to a person’s ordinary job, overtime work, client dinner, housework, parenting, and nursing care.

POMS2-A is the tool for assessing transient, distinct mood states. The full version of POMS2-A consists of 65 questions and T-scores of 6 mood clusters—Anger-Hostility (AH), Confusion-Bewilderment (CB), Depress-Dejection (DD), Fatigue-Inertia (FI), Tension-Anxiety (TA), and Vigor-Activity (VA). Total Mood Disturbance (TMD) is determined by adding the T-scores of AH, CB, DD, FI, and TA—negative mood states—and subtracting the VA T-score—positive mood state. Friendliness (F) is considered separately from other mood states.

The primary outcome was the 8-week change in VAS-1 from the baseline. The secondary outcomes were the 8-week changes in the rest of the VAS values (VAS 2–4). Also, the changes in POMS2-A T-scores of Fatigue-Inertia and Vigor-Activity from the baseline were included as secondary outcomes. VAS and POMS2-A questionnaires were collected at every visit with 4-week intervals. The clinical visits at baseline, week 0, and week 8 were from May to July for all participants within 17 days difference for each visit.

Common Terminology Criteria for Adverse Events (CTCAE) v4.0 was used to count the possible adverse events during the study.

### Statistical analysis

Judging from the expected reduction in VAS-1 of 18 and a standard deviation of 24, the sample size was estimated to be 70. The assumed statistical power was 85% with a two-sided type I error of 5%. The normal distributions of all major outcomes, except for the 8-week change in VAS-3, were confirmed by a histogram curve. The 8-week changes were compared between the groups by Student’s t-test. In the case of the 8-week change in VAS-3, which did not show a normal distribution, the Mann–Whitney *U* test was applied. The pairwise data (pre- and post-intervention comparisons) were analyzed by paired *t* test. Two-way repeated measures analysis of variance (ANOVA) using tablet intake ratio as covariate was applied to assess the time × group interaction effect for primary and secondary outcomes. In these analyses, the data were logarithmically transformed in cases of non-normal distributions (AH, DD, TA, and TMD) Data were analyzed as a full-analysis set (FAS), and the multiple-imputation method repeated 20 times to generate the dataset was used to fill in missing data. Fisher’s exact test was applied for categorical variables to determine the difference in adverse events between the groups. Statistical analyses were performed using the SPSS (Statistical Package for the Social Sciences) Statistics 22. Data are expressed as the mean ± SD, and *P* < 0.05 was considered significant.
